# Expression of miR-148b-3p is correlated with overexpression of biomarkers in prostate cancer

**DOI:** 10.1590/1678-4685-GMB-2018-0330

**Published:** 2020-03-06

**Authors:** Eliakym Arámbula-Meraz, Fernando Bergez-Hernández, Emir Leal-León, Enrique Romo-Martínez, Verónica Picos-Cárdenas, Fred Luque-Ortega, Jose Romero-Quintana, Marco Alvarez-Arrazola, Noemí García-Magallanes

**Affiliations:** 1 Universidad Autónoma de Sinaloa, Facultad de Ciencias Químico Biológicas, Laboratorio de Genética y Biología Molecular, Culiacán, Sinaloa, Mexico.; 2 Universidad Autónoma de Sinaloa, Programa de Posgrado en Ciencias Biomédicas, Culiacán, Sinaloa, Mexico.; 3 Universidad Politécnica de Sinaloa, Unidad Académica de Ingeniería en Biotecnología, Laboratorio de Biomedicina y Biología Molecular, Mazatlán, Sinaloa, Mexico.; 4 Universidad Autónoma de Sinaloa, Facultad de Medicina, Laboratorio de Genética, Culiacán, Sinaloa, Mexico.; 5 Instituto Mexicano del Seguro Social, Culiacán, Sinaloa, Mexico.; 6 Alvarez & Arrazola Radiólogos, Mazatlán, Sinaloa, Mexico.

**Keywords:** Gene expression, biomarker, miRNAs, correlation, prostate cancer

## Abstract

Prostate cancer (PCa) is one of the leading causes of death among men. Genes such as *PCA3*, *PSA*, and *Fra-1* are suggested to serve as potential tools for the detection of PCa, as they are deregulated during this pathology. A similar event occurs with small non-coding RNAs, called miRNAs, specifically miR-195-5p, miR-133a-3p, and miR-148b-3p, which were analyzed in a Chinese population and suggested to be possible candidates for PCa diagnosis. We evaluated the expression levels of three miRNAs and three genes in tissue samples of PCa and benign prostate disease, such as benign prostatic hyperplasia, or prostatitis, in order to determine their potential as candidates for PCa detection. Our results showed a statistically significant overexpression of 279-fold increase in *PSA* levels and a 1,012-fold increase in *PCA3* levels in PCa patients compared to benign prostate disease patients (*p* = 0.001 and *p* = 0.002, respectively). We observed a positive correlation between the expression of miR-148b-3p and the expression of *PSA* and *PCA3* genes, two established biomarkers in PCa. The expression of miR-148b-3p was not related to clinical characteristics, such as age and weight, as observed for the other miRNAs analyzed, suggesting its potential as a biomarker for detection of this pathology.

## Introduction

Prostate cancer (PCa) is the fourth most common cancer worldwide and the second most common type of cancer diagnosed in men. It is also recognized as the most frequent malignant tumor in men older than 50 years (GLOBOCAN, 2012). Several genes involved in the progression of PCa, such as the *Prostate Cancer Associated 3* (*PCA3*), the *Fos-related antigen 1* (*Fra-1*) and the *Prostate Specific Antigen* (*PSA*), present different expression levels in individuals with and without cancer. In this context, *PCA3* is one of the most reported genes (Bussemakers *et al.*, 1999; Floriano-Sánchez *et al.*, 2009). This gene is exclusively expressed in prostatic tissue and is found highly overexpressed in patients with PCa. For this reason, the *PCA3* gene is suggested as an interesting biomarker for this pathology (Xue *et al.*, 2014).

Recent studies have shown that the *Fra-1* gene is also deregulated in patients with PCa. *Fra-1* is a proto-oncogene that encodes a transcription factor with a central role in the regulation of several biological processes, including cell proliferation, differentiation, transformation, and inflammation. Thus, *Fra-1* is also in the spotlight for its potential as a biomarker in PCa (Adiseshaiah *et al.*, 2005; Zhu *et al.*, 2014).

Current biomarkers for the detection of PCa have low specificity and sensitivity, as is the case of PSA, a protein that is not specific for this malignancy, and that may be elevated by different pathologies (Daniyal *et al.*, 2014). It is important to mention that most studies on PSA are directed to quantifying serum PSA levels, but only a few have focused on measuring gene expression. These studies suggested that *PSA* gene expression may be used in combination with other biomarkers to offer a better diagnosis (ACS, 2012; Coelho *et al.*, 2015).

Small non-coding RNAs (known as microRNAs or miRNAs) in charge of regulating gene expression have been reported deregulated in several pathologies (McDonald *et al.*, 2017). In PCa studies, an amounting evidence has demonstrated the importance of miRNAs for this pathology. This is especially true for miR-195-5p, miR-133a-3p, and miR-148b-3p as their deregulation causes the inhibition of proliferation, migration, and invasion of PCa cells (Xu *et al.*, 2009; Li *et al.*, 2011; Tao *et al.*, 2012; Kojima *et al.*, 2012; Dai and Grant, 2015; Wu *et al.*, 2015). MiR-195-5p plays a regulatory role in migration, invasion, proliferation, epithelial-mesenchymal transition, angiogenesis, and metastasis of tumor cells by targeting the 3’UTR sequence of *RPS6KB1*, *FGF2*, *Fra-1*, and *BCOX1* genes (Cai *et al.*, 2015; Liu *et al.*, 2015; Wu *et al.*, 2015; Guo *et al.*, 2015). On the other hand, miR-133a-3p deregulation is suggested to be a key step in oncogenesis and progression of PCa due to regulation of the *PNP* gene (Kojima *et al.*, 2012). Lastly, miR-148b-3p is associated with various carcinogenic genes, and its expression level is found deregulated in several types of cancer. In general, downregulation of this miRNA relates to high-grade tumors. More importantly, miR-148b-3p is suggested as an indicator to distinguish malignant from benign prostate disease (Watahiki *et al.*, 2011; Walter *et al.*, 2013).

Clinical evidence demonstrates that early diagnosis of PCa is determinant to improve the treatment outcome. Therefore, the aim of this study was to analyze the expression and correlation of different miRNAs (miR-195-5p*,* 133a-3p and 148b-3p), genes (*Fra-1*, *PSA*, and *PCA3*), and clinicopathological characteristics (age, body weight, and serum PSA) to be used independently or in combination as biomarkers for early diagnosis of PCa.

## Subjects and Methods

### Patient recruitment

Our study group consisted of 19 patients: 13 diagnosed with PCa and 6 with benign prostate disease (BPD). The BPD group included men with benign prostatic hyperplasia or prostatitis. All PCa and BPD patients met the inclusion criteria: Mexican men older than 18 years with a diagnosis for PCa or BPD confirmed by histopathology, neither receiving chemotherapy nor radiotherapy, and not presenting any other type of cancer. All patients were recruited either from the Mexican Social Security Institute (MSSI) or from the Alvarez and Arrazola Radiologists Clinic, both located in Sinaloa, Mexico, from August 2016 to September 2017. Clinicopathological data (age, weight, height, serum PSA, and other diseases) were collected through direct questionnaire and hospital or clinic database and the Gleason score was provided by a pathologist. All patients granted approval by signing an informed consent that was previously reviewed and approved by the Ethics and Research Committee of the MSSI and Alvarez and Arrazola Radiologists Clinic.

### Tissue samples and RNA extraction

Tissue samples were obtained through transrectal biopsy and were used for RNA extraction. Total RNA, including miRNAs, was isolated using the miRNeasy kit (Qiagen, Hilden, Germany) according to the manufacturer’s protocol. The concentration of isolated RNA was measured with the assistance of the GENESYS 10S UV-Vis Spectrophotometer (Thermo Scientific TM).

### Relative expression of miRNAs

Reverse transcription (RT) was performed from 10 ng of total RNA with the TaqMan Advanced miRNA cDNA Synthesis kit (Applied Biosystems,). Quantitative estimations of miR-195-5p, miR-133a-3p*,* and miR-148b-3p were performed by real-time polymerase chain reaction (RT-PCR) method using TaqMan^®^ MicroRNA Assays (Applied Biosystems). We normalized the expression of our miRNAs of interest using miR-16-5p as the reference gene. Briefly, we followed the procedure described in Andersen *et al.* (2004) to identify the most appropriate gene based on expression stability. The NormFinder Software allowed us to identify miR-16-5p as the best candidate (stability value = 0.013). The results are presented as the ratio of the number of copies of a given gene to that of the reference gene. To obtain data about the relative expression of the miRNAs, we used the ΔΔCq method (2^-DDCq^ algorithm) (Livak and Schmittgen, 2001).

### Relative expression of *Fra-1*, *PSA*, and *PCA3*

RT was performed from 1 μg of total RNA with ImProm-II^TM^ Reverse Transcriptase (Promega Corporation, Madison, WI, USA) and random primers. Quantitative estimation of *Fra-1*, *PSA*, and *PCA3* transcripts were performed by real-time PCR method with TaqMan^®^ Assays (Applied Biosystems) on StepOnePlus^TM^ Real-Time PCR system (Applied Biosystems). We used *beta actin* (*Act-*β) as reference gene. The reaction conditions consisted of enzyme activation at 95 °C for 20 s, followed by 40 cycles of 95 °C for 1 s and 60 °C for 20 s. All samples were assessed in technical duplicates. If Cq (quantification cycle) values obtained from technical duplicates were in discrepancy, the sample was reassessed. To obtain data about relative gene expression we used the algorithm of Livak and Schmittgen (2001).

### Statistical and correlational analysis

The Student’s *t*-test and Mann-Whitney U-test (when appropriate) were used to compare differences between continuous variables. Due to small sample size, the Fisher’s exact test was used to compare differences between dichotomous variables. Pearson and Spearman tests (when appropriate) were used to calculate the correlation coefficient between the following variables: expression of miRNAs, expression of *Fra-1*, *PSA*, and *PCA3*, serum PSA, age, and body weight. All these variables were contrasted among each other to observe relationships.

The Statistical Package for the Social Sciences (SPSS, Inc., Chicago, IL,) version 20 software was used for all statistical calculations. Results with a *p*-value < 0.05 were considered statistically significant.

## Results

### Clinicopathological characteristics

Our study population had an average age of 67.11 ± 7.04 years. In PCa patients the average age was 67.54 ± 7.95 years and in BPD patients, 66.67 ± 5.09 (*p* = 0.810). Table 1 shows the results of the clinicopathological characteristics analysis. Evaluation of the body mass index (BMI) yielded an average of 25.54 ± 1.56 in our total population (Table 1). The results showed no statistical significance (*p* = 0.359) between groups.

**Table 1:** Clinical characterization of prostate cancer (PCa) and benign prostate disease (BPD) groups.

Variable	PCa (n=13)	BPD (n=6)	*p*
Mean age (years)	67.54 ± 7.95	66.67 ± 5.09	0.81
Mean BMI	25.74 ± 1.69	24.78 ± 0.79	0.359
PCa family history (yes) %	23.1	66.7	0.046^*^
Mean PSA (μg/L)	102.78 ± 214.90	17.15 ± 1.62	0.513

* Statistically significant value. BMI: body mass index; PSA, Prostate specific antigen.

An exhaustive medical examination of our groups revealed that 10.5% of the subjects presented prostatitis, 15.8% exhibited dyslipidemias and other neoplasms, and 36.8% suffered from diabetes and hypertension (Table 1). However, we found no statistical difference (*p*e = 0.233) between groups.

Concerning family history of cancer, we found that 15.4% of our patients with PCa had first-degree relatives who suffered from the same disease, and 7.7% mentioned relatives with breast cancer, for a total of 23.1% of patients with at least one family member affected by some type of cancer. Regarding our BPD group, 33.3% had no family history with malignant neoplasms, and 66.7% had first-degree relatives with PCa (Table 1).

**Figure 1:**
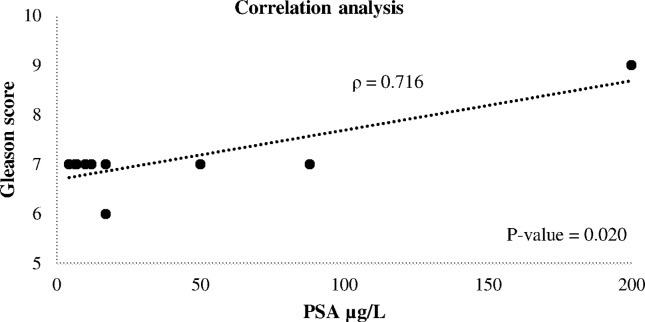
Correlation between levels of serum PSA and Gleason score. The correlation coefficient of 0.716 with p-value < 0.05 indicates that serum PSA was moderately correlated with Gleason score.

Classifying our patients using the Gleason score, we observed that in the PCa group, 9.1% were classified as 6 (3+3), 36.36% as 7 (3+4), 36.36% as 7 (4+3), and 18.18% as 9 (5+4), corresponding to low, low-intermediate, intermediate-high, and high aggressiveness, respectively. Patients with a Gleason score of 6 (3+3), 7 (3+4), 7 (4+3), and 9 (5+4) exhibited mean levels of PSA of 16, 8.83, 39.82, and 200 μg/L, respectively. Performing a Spearman correlation test, we observed that there was a relationship between the level of serum PSA and the aggressiveness of the tumor (*p* = 0.020, ρ = 0.716) (Figure 1).

### Relative gene expression levels

**Figure 2:**
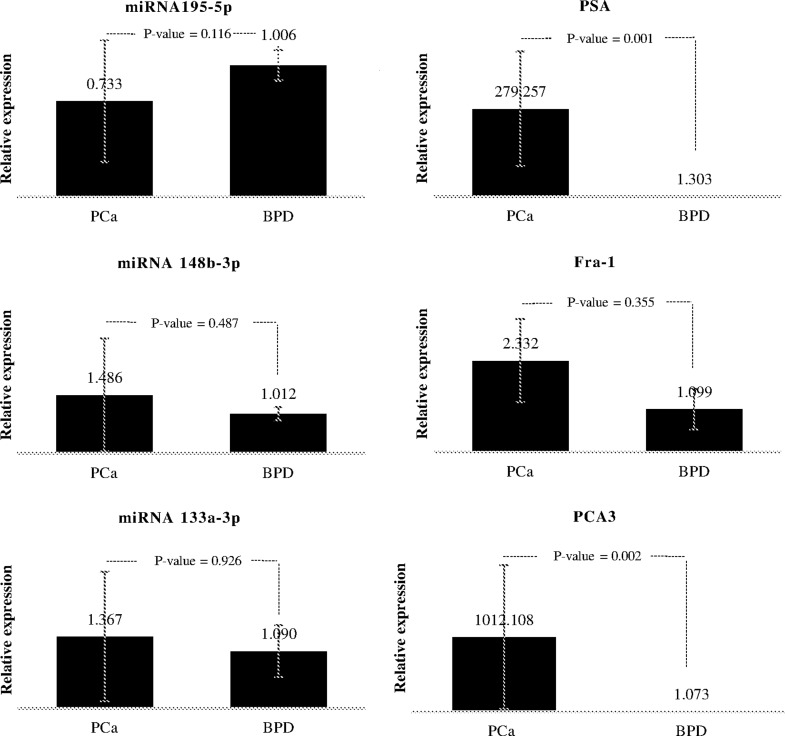
Expression levels of miRNAs, PSA, PCA3, and Fra-1 genes. Real-time PCR exhibited similar expression levels of the three miRNAs (left column) and Fra-1 gene (mid-right) in PCa and BPD tissue samples. Expression levels of PCA3 (bottom-right) and PSA (top-right) genes were significantly higher in PCa than in BPD samples. Error bars indicate ± SD.

For miR-195-5p, 148-3p, 133a-3p, and the *Fra-1* gene, we found no statistically significant difference in expression levels in prostatic tissue between men with PCa and BPD (*p* = 0.116, *p* = 0.487, *p* = 0.926, and *p* = 0.355, respectively) as shown in Figure 2. However, our results showed a statistically significant overexpression of 279-fold increase in the *PSA* levels and a 1,012-fold increase in the *PCA3* levels of PCa patients compared to BPD (*p* = 0.001 and *p* = 0.002, respectively) (Figure 2).

### Correlation analysis

**Figure 3:**
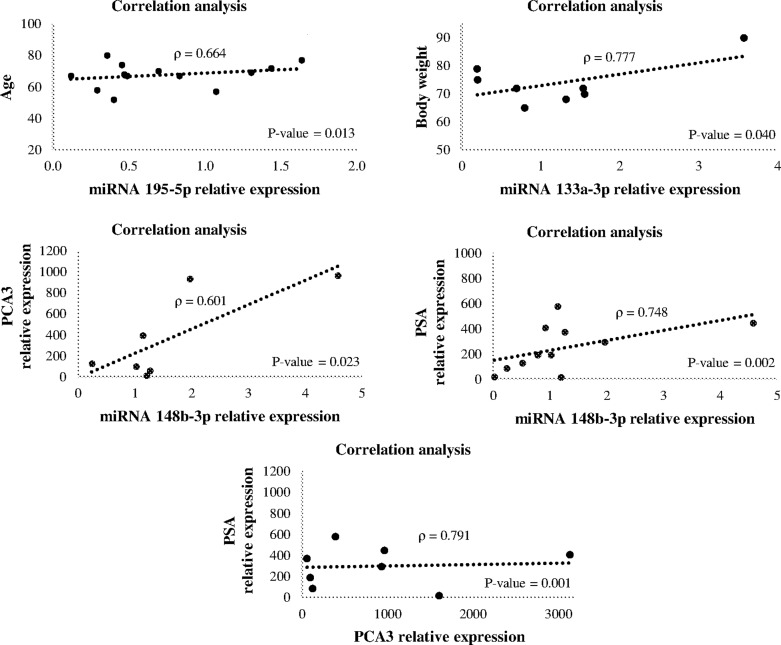
Correlation analysis between expression levels of miRNAs, clinicopathological characteristics, PSA, and PCA3 genes. The correlation coefficient of 0.664 with p-value < 0.05 indicates that miR-195-5p expression was moderately correlated with age in PCa patients. Also, there was a strong correlation between body weight and miR-133a-3p expression with a correlation coefficient of 0.777 (p < 0.05). There was also a moderate correlation between miR-148b-3p and PSA or PCA3 expressions with a correlation coefficient of 0.601 and 0.748, respectively. Correlation coefficient of 0.791 (p < 0.05) indicated a strong correlation of PCA3 with PSA expression.

In the correlation analysis, we observed a relationship between miR-195-5p expression and the age of patients with PCa (p = 0.013) with a correlation coefficient of 0.664. In addition, the expression of miR-133a-3p correlated with an increase in body weight with a correlation coefficient of 0.777 (p = 0.040). Regarding the expression of miR-148b-3p, a positive relationship was observed when compared with the expression levels of PCA3 and PSA genes, with correlation coefficients of 0.601 and 0.748, respectively (p = 0.023 and p = 0.002). Lastly, PSA and PCA3 expression levels showed a strong correlation with a coefficient of 0.791 (p = 0.001) (Figure 3).

## Discussion

Deregulation in gene expression, that is, an increase or decrease in the production of mRNA, is a frequent event in PCa. Thus, the analysis of genes that are associated with the development and progression of PCa offers a new strategy for specific and early detection, addressing the most important problem in PCa and avoiding unnecessary biopsies.

We analyzed the expression of *PCA3* and *PSA*, two genes commonly reported in this pathology due to their clinical value as biomarkers. Our results showed an overexpression of *PCA3* in tissues from PCa patients, similar to previous reports where its expression level was evaluated in different body fluids and tissues (Bussemakers *et al.*, 1999; Li *et al.*, 2018). Nonetheless, only a handful of analyses has been conducted in the Mexican population (Floriano-Sánchez *et al.*, 2009), highlighting the necessity to investigate the expression of *PCA3* and *PSA* in these individuals. Moreover, as previously reported by de la Taille *et al.* (2011), we observed a correlation between the expression of *PCA3* and the Gleason score; a high level of *PCA3* was related to values ??above 7 on the Gleason score, suggesting that the expression level of this gene could serve as an indicator of aggressiveness in Mexicans as well (de la Taille *et al.*, 2011).

PSA is a widely used protein for PCa detection, due to its ability to identify abnormalities in the prostate. However, it is not specific for PCa. In this regard, analyses of the *PSA* gene in prostatic tissue could provide more accurate information about the presence of this disease. Thus, we analyzed the relationship between the expression of the *PSA* gene and the expression of five other genes (*Fra-1*, *PCA3*, miR-195-5p, miR-133a-3p, and miR-148b-3p) in prostatic tissue, as well as the protein levels in the serum. Interestingly, we observed no relationship between the expression of the *PSA* gene and the concentration of serum PSA. This can be due to different factors. In 1995, it was discovered that *PSA* is expressed not only in the prostate but also in various tissues and structures, such as the periurethral glands, perianal glands, and apocrine sweat glands (Graves, 1995), showing that serum PSA is not prostate-specific. This makes it necessary to search for alternative biomarkers for the detection of PCa.

In this study we investigated different genes that are confirmed to be of importance in PCa, such as *Fra-1*. *Fra-1* is a pro-oncogenic and pro-angiogenic gene known for activating the IL-6/JAK/Stat3 signaling pathway, and for promoting the release of MMP-9, MMP-2, VEGF, and TGF-β in breast and lung cancer (Adiseshaiah *et al.*, 2005; Luo *et al.*, 2010). In addition, *Fra-1* has been shown to be involved in regulating growth, migration, and invasion in two different prostate cancer cell lines, *DU145* and *PC3* (Wu *et al.*, 2015). However, these findings have yet to be confirmed in PCa patients, as *in vitro* results not always translate to humans. We found this to be true in Mexican PCa patients, in which *Fra-1* was observed at similar expression levels as the control group, suggesting that in Mexican men, this gene may not serve as a positive indicator of PCa.

To explore the potential of different molecules as biomarkers, we also evaluated the expression levels of three miRNAs suggested to be involved in this pathology. We decided to investigate miR-195-5p, miR-133a-3p, and miR-148b-3p. With a little less than a half a decade of study, these three miRNAs became rapidly associated with cancer. Underexpression of miR-195-5p and 133a-3p is related to proliferation, invasion, and migration in PCa cells and, in the case of miR-195-5p, also metastasis. miR-195-5p mediates cellular processes through targeting the *fibroblast growth factor 2 (FGF2)* and *BCOX1* genes (Guo *et al.*, 2015; Liu *et al.*, 2015). On the other hand, miR-133a-3p regulates genes such as *EGFR*, *CASP9*, and *IGF1R* (Tao *et al.*, 2012; Pashaei *et al.*, 2017). These genes have already been reported to be implicated in different processes of PCa, such as apoptosis, metastasis, and androgen-independence (Wu and Yu, 2014; Day *et al.*, 2017; Yilmaz *et al.*, 2017). miR-148b-3p ha, so far,s not been studied in depth in PCa. However, it is known to play a role in gastric, lung, and bladder cancer through the regulation of the Wnt, MAPK, and Jak-STAT signaling pathways, which are involved in PCa (Luo *et al.*, 2015; Huang, 2016). The Wnt family plays an important role in cell proliferation and differentiation (Angers and Moon, 2009, Whyte *et al.*, 2012). It has been observed that Wnt signaling is related to the development of prostate cancer (Murillo-Garzón and Kypta, 2017). The MAPK pathway has been reported to be involved in the growth and metastasis of PCa (Bradham and McClay, 2006; Wagner and Nebreda, 2009) and the JAK/STAT pathway has been observed upregulated in PCa. Furthermore, by suppressing JAK/STAT3 signaling, cell growth is suppressed and apoptosis is promoted (Aalinkeel *et al.*, 2010; Aghaee-Bakhtiari *et al.*, 2015).

Our results showed, that when compared to BPD patients, PCa patients expressed 27.1% lower miR-195-5p levels, 25.4% higher miR-133a-3p levels, and 46.8% higher miR-148b-3p levels; however, none of these results were statistically significant. These results differ from previous reports conducted on the same set of miRNAs carried out in Chinese populations (Guo *et al.*, 2015; Liu *et al.*, 2015; Wu *et al.*, 2015), whereas ours was conducted in Mexicans. When analyzing the ancestry of Mexican men, Martínez-Cortés *et al.* (2012) showed that in the Sinaloa, region, where our patients were from, European ancestry is of 63%, whereas and the Asian is as low as 1% This could explain the discrepancy between the previous and our study.

It is important to identify whether clinicopathological characteristics are capable of modifying the expression levels of the miRNAs involved in our study. Hence, correlation analyses were performed to determine elationships between the variables. We observed an association between the age of patients and expression of miR-195-5p, suggesting that levels of this transcript increase over time. There is limited information about miR-148b-3p in PCa and, although we did not observe a significant difference in expression between men with PCa and BPD, correlations were identified between miR-148b-3p and the expression levels of *PCA3* and *PSA,* two genes highly related to PCa. The observed relationship demonstrates the importance of studying alternative molecules involved in the control of gene expression in PCa to understand the control mechanisms underlying this pathology, improve detection methods, and propose new therapeutic approaches.

Here, we present evidence that *PCA3* and *PSA* expression levels in tissue are suitable for differentiating between men with benign or malignant PCa disease. We observed a correlation between the expression of miR-148b-3p in tissue with the overexpression of *PSA* and *PCA3* genes, which are established biomarkers in PCa. The expression of this miRNA was not related to variables such as age and weight, as observed in the other miRNAs analyzed, suggesting its potential as a biomarker for this pathology.
